# Metacognition About Collaborative Learning: Students’ Beliefs Are Inconsistent with Their Learning Preferences

**DOI:** 10.3390/bs14111104

**Published:** 2024-11-16

**Authors:** Yunfeng Wei, Nicholas C. Soderstrom, Michelle L. Meade, Brandon G. Scott

**Affiliations:** Department of Psychology, Montana State University, P.O. Box 173440, Bozeman, MT 59717-3440, USA; nicholas.soderstrom@montana.edu (N.C.S.); mlmeade@montana.edu (M.L.M.); brandon.scott2@montana.edu (B.G.S.)

**Keywords:** metacognition, collaboration, strategy, collaborative memory, learning

## Abstract

Collaboration plays an important role in educational contexts. However, little is known about students’ metacognitive beliefs about collaboration. The present study used an online survey to investigate students’ beliefs toward group study/recall, their studying preferences, strategies they use when studying individually and in groups, and important characteristics of their group members. Results indicate that, although students generally perceive collaboration as beneficial, they prefer individual study, indicating that their beliefs are inconsistent with their learning preferences. Students report social learning as the primary reason for collaborative benefits but prefer to study alone to minimize distraction and increase personal accountability. Further, they use different strategies when studying individually or in a group. When studying individually, students most frequently report re-reading their notes. However, when studying in groups, students most frequently use strategies emphasizing collaboration and interaction, such as quizzing each other. Also, students prefer to work with group members who are focused, motivated, and hard working. Students’ beliefs, preferences, and favored characteristics of group members are related to their frequency of using study groups. Students’ metacognitive beliefs about collaboration have implications for theories of self-regulated learning and better use of collaboration in educational contexts.

## 1. Introduction

Students often work together in educational settings. For example, they collaborate on group projects and group discussions, and they rely on study groups to prepare for exams. Such group activities are common in education, yet the effects of collaboration on cognition are mixed [1,2]. Importantly, there is limited research on students’ metacognitive beliefs and preferences regarding collaboration. Metacognitive beliefs are an important aspect of learning because students rely on their metacognition to monitor their learning and select appropriate strategies [3]. In the present study, we examine students’ perceptions of collaborative study, collaborative testing, their use of study groups, the strategies they use when studying individually and in groups, and which characteristics of their fellow group members students believe are most important for successful collaboration.

## 2. Collaborative Cognition

Many researchers encourage instructors to use collaboration in educational settings because learning in groups can improve students’ motivation, engagement in classes, reasoning, and problem-solving skills [4]. Other researchers have claimed that students who recall information in groups are subsequently better able to recall information individually, a phenomenon called post-collaborative benefits [5,6,7]. Consequently, many cognitive and educational researchers regard collaborative learning as a powerful pedagogical tool and, therefore, recommend its use in educational contexts.

In spite of its many benefits, collaboration is not always beneficial in terms of memory performance. First, individuals recalling information with other group members may suffer from collaborative inhibition, or worse recall in groups [7,8]. Second, individuals recalling with others may learn misinformation from group members, which is called the social contagion effect [9,10]. Finally, recalling with other individuals can lead to forgetting. Research on socially shared retrieval-induced forgetting demonstrates that listening to someone recall information can lead to forgetting related information [11].

Given the mixed results of collaboration on learning and memory, it is important to understand student perceptions about collaboration. Previous metacognitive research in collaboration has focused on perceptions of group-level processes such as coordinating information and expertise, delegating tasks, developing strategies, etc. [12]. In contrast, the current study is focused on understanding student perceptions of how collaboration influences their learning and memory during study groups and how such perceptions relate to their study behaviors and preferences. Further, given that there are situations in which collaboration is relatively more beneficial or harmful, it is important to understand students’ beliefs about the characteristics of successful collaborative groups.

## 3. Metacognition

Metacognition refers to people’s understanding of their own cognitive processes, as well as one’s knowledge and understanding of how learning and memory operate [13,14]. Metacognitive research has demonstrated that students rely on metacognition to regulate their learning, and metacognition can directly affect subsequent behavior [15]. For example, individuals’ judgments of learning (JOL)—that is, their subjective assessments of their own learning—are linked to self-regulated learning: As judgments of learning become more accurate, students make better study choices [16]. Students’ metacognitive beliefs about collaboration, then, have important implications for theories of self-regulated learning because they can inform if and how students rely on others to regulate their learning.

Given the importance of metacognition to self-regulated learning, it is noteworthy that students’ metacognitive judgments do not always correspond to their cognitive performance [17,18]. For example, Blasiman et al. [19] demonstrated that students’ intended study behaviors may not correspond to their actual study behaviors by showing that students intended to study twice as long as they did. The dissociation between metacognition and cognition is especially relevant to the current study because students’ beliefs about study groups may or may not relate to their actual use of study groups and/or how study groups influence their learning.

Theories of self-regulated learning also suggest that students rely on metacognitive beliefs to select study strategies [3]. Again, there is a dissociation between metacognition and cognition in the strategies students use to study, as students often prefer to use non-optimal strategies. For example, Karpicke et al. [20] surveyed college students to investigate the strategies students prefer when studying. They found that students most frequently re-read notes to prepare for exams [21,22]. Further, students are rarely aware of the usefulness of testing as a powerful learning strategy, though the testing effect—the memorial benefit of retrieving information from memory—has been replicated many times in a wide variety of contexts [23,24]. Together, this research suggests that students rely on their metacognitive beliefs about learning to select strategies they believe will benefit their learning in individual contexts. Importantly, little is known about the strategies used in collaborative groups, and it remains unknown if and how students’ preferred strategies differ across individual and collaborative study contexts.

## 4. Students’ Perceptions of Collaboration

To our knowledge, only a handful of studies have examined participants’ metacognitive beliefs about collaboration. In the current study, we replicate and extend previous research to examine the following factors that we believe can best inform theory and practical applications: (1) the relationship between students’ beliefs about collaboration, their actual use of collaborative groups, and their study preferences; (2) the distinction between collaborative study and collaborative recall; (3) a direct comparison of individual strategies and strategies used in collaborative groups; (4) characteristics associated with effective group members; and (5) differences in metacognitive beliefs between students who frequently and rarely use study groups.

### 4.1. Beliefs, Frequency of Collaboration, and Preferences

Several studies have surveyed students’ beliefs about collaboration and their use of collaborative groups, e.g., [25,26]. These studies demonstrate that students generally believe that recalling in groups is more effective than recalling individually (see too related research showing that individuals view collaboration positively, regardless of whether or not performance is better in the collaborative group [27] or worse in the collaborative group [28].

However, the literature on students’ actual use of collaborative groups is more mixed. Wissman and Rawson [25] reported that 41% of students spend at least one hour per week in collaborative groups, and McCabe and Lummis [29] reported that 78% of students study in a group at least once per semester. In contrast, Rybczynski and Schussler [26] reported that students rarely used study groups (55% never used study groups, and 22% tried but quit over the course of the semester). Students’ preferences for individual or group study are also inconsistent. McCabe and Lummis reported that nearly half of students prefer to study alone (47.6%) rather than study collaboratively, while Rybczynski and Schussler reported that only a small proportion of students (5 out of 246) prefer to study alone, but students became more likely to prefer to study alone and less willing to participate in collaborative study as the semester progressed. Considered together, existing research suggests that students generally believe that collaboration is beneficial, but it is unclear how frequently they engage in collaborative learning or if they prefer collaborative learning over individual learning.

In the current study, we directly test the relationship between beliefs, frequency, and preference within a single study. Consistent with previous findings demonstrating that metacognition is often inconsistent with cognitive performance [18,19], we hypothesized that students would have a positive attitude toward study groups but rarely use study groups and prefer to study alone, indicating their beliefs are inconsistent with their actual and preferred behaviors.

### 4.2. Group Recall and Group Studying

Wissman and Rawson [25] asked students questions specific to collaborative study and collaborative testing. Interestingly, only 25% thought studying in a group was more beneficial than studying alone, but regarding testing specifically, 51% said they learn more when testing in a group vs. alone. When asked why testing is more effective in groups, the most common explanation (22% of participants) was that collaborative testing helps students figure out what they do not know because they cannot look at the answer. These results highlight the important distinction between collaborative studying and collaborative testing and, importantly, demonstrate that students differentiate study and testing dimensions of collaboration. However, this study did not fully code participants’ qualitative responses, and so it remains unknown why students believe collaboration has different effects on studying and testing.

In the current study, we ask students open-ended questions about why they believe studying/recalling in groups/individually is more effective. Our hypotheses here are exploratory, as we are seeking to further characterize students’ metacognitive beliefs. Nonetheless, we hypothesized that when students choose to study or recall in groups and study or recall individually, their underlying reasons would be different.

### 4.3. Strategies Used When Studying Individually and in Groups

Previous research has established that undergraduate students report re-reading notes as a preferred strategy to study for exams individually [20,21,22]. Relatively less is known about students’ preferred strategies when studying for exams in groups. Wissman and Rawson [25] found that 79% of students reported quizzing each other. Rybczynski and Schussler [26] reported the most common strategy used in collaborative groups (98% of students) was discussing or clarifying notes with group members. Finally, McCabe and Lummis [29] reported that the most frequently used strategy in collaborative groups was to ask questions.

In these studies, participants were presented with several strategies and asked to check all the strategies they use during collaborative learning [25,26] or to rate how frequently they engaged in each strategy [29]. The three studies presented different strategy options for students to select from, so it is difficult to directly compare results. However, using an open-ended question asking which strategies students used in groups, McCabe and Lummis reported that the most frequently listed group strategy was to work on practice problems, followed by discussing course materials, quizzing or testing each other, and asking questions, results that are broadly consistent with the forced choice data. Importantly, however, these studies did not ask students about their preferred strategies in groups, and none of these studies included an individual comparison condition.

In the current study, we extend this research to ask open-ended questions about the strategies students use during collaboration, and critically, we also ask them to rank order their preferred/most used study strategies [20]. Further, we include a comparison of individual study strategies to determine how the frequency/rating of strategies differs across individual and group study. Because students highlight collaboration in study groups [25,26,29], we hypothesized that students use similar strategies when studying in groups compared to studying individually but that they prioritize more interactive strategies when studying in groups because, unlike studying individually, studying in groups affords such interactive strategies.

### 4.4. Characteristics of Other Group Members

Another exploratory element of this study was to examine what characteristics students believe are important of their group members. Rybczynski and Schussler [26] provided a qualitative analysis of student beliefs about collaboration. Students reported that group composition is important, equal contribution from group members is vital, study groups often lack focus and are not productive, and that social learning has inherent value. The surveys conducted throughout the semester were administered immediately following class exams, and so students’ perceptions of any collaboration prior to the exam were likely influenced by their exam performance. Nonetheless, these data suggest that students’ metacognitive beliefs about collaborative study groups depend at least partially on how the group performs. This is relevant to our interest in better understanding what characteristics students believe contribute to successful study groups.

In the current study, we replicate and extend the results of Rybczynski and Schussler [26]. Consistent with their results showing that students believe that focus, equity, and productivity are the major concerns of students studying in groups, we hypothesized that such characteristics would also be important to our participants. However, given our open-ended format, we were able to identify other characteristics that students found important of their group members.

### 4.5. Differences Between Students Who Frequently and Rarely Use Study Groups

As previous studies show [25,26,29], some students frequently use study groups, while some others rarely or never use study groups. Further, there is some evidence that students who frequently use study groups have different metacognitive beliefs compared to those who rarely use study groups. Wissman and Rawson reported that 71% of students who rarely studied in groups (less than 1 h per week) believed individual study was more beneficial, while only 40% of students who frequently studied in groups (more than 1 h per week) believed individual study was more beneficial. Likewise, McCabe and Lummis reported that students who frequently use group study use more effective strategies during group study. In the current study, we replicate and extend these results to examine how experience influences metacognitive beliefs about collaboration. That is, besides surveying students’ (1) beliefs, (2) preferences, (3) strategies, and (4) group member features, we split participants into those who frequently use groups and those who rarely use groups to further examine how students’ previous study group experiences are related to their metacognition about study groups.

Finally, we note that this study was conducted during the COVID-19 pandemic. Research suggests that social distancing and isolation during the pandemic significantly impacted students’ well-being [30] and students’ learning environments as many classes moved online [31]. Further, Rosenfield et al. [32] argue that group processes and interpersonal relationships are among the psychological phenomena most likely to show lasting impacts from the COVID-19 pandemic. As such, this study is unique in examining student perceptions of collaboration during the pandemic and serves as a time point that can help future researchers examine if/how student perceptions about collaborative learning change in the coming years as any effects of the pandemic settle.

## 5. Method

### 5.1. Participants

Two hundred and two students participated in the online survey as a part of the Introduction to Psychology course credits. The mean age of participants was 20.10 years, and the majority of participants were first-year students (121 first year, 46 second year, 24 third year, nine fourth year, and two others). The sample included 81 males, 118 females, and three third gender, and most of the participants were White (93%). The sample size in the present study was determined in accordance with previous studies that examined the strategies students use [20,21,25,26]. Blasiman et al. [19] recommended 200 or more participants for survey research of student strategies; our sample size exceeded this recommendation.

The data were collected in the spring semester of 2021 during the COVID-19 pandemic. At this time, Montana State University was in the middle of its second semester of blended learning (students alternated between attending class in person and online), and students had experienced disruptions to their personal and academic lives. As a result, the sample used in the current study offers a unique and important perspective on students’ metacognitive beliefs about collaboration during the pandemic.

### 5.2. Materials and Procedure

Survey questions were developed locally or adapted from previous research [20,26,33] (see Appendix A for the full survey). The survey included questions regarding participants’ beliefs about studying in groups versus studying individually and their study behaviors, including the following: “How often do you use study groups?”; do you prefer to study for exams in a group or individually?; which do you think is more effective—studying individually or studying in groups?; and which do you think is more effective—recalling individually or recalling in groups? Asking participants which is more “effective” is consistent with past research on the strategies that students perceive as most effective for learning [33]. For these questions, we used a forced-choice format rather than a Likert scale to avoid any neutral or ambiguous responses. That is, we wanted participants to directly compare these two methods and select which one they thought was more effective. We also asked participants to answer why they thought individual or collaborative study/recall was more effective, and why they preferred to study individually or collaboratively. The “Why” questions were open-ended and helped verify that participants interpreted the question as effective for learning. Furthermore, participants were asked to list strategies they used when studying individually and in groups, from the most frequently used to the least frequently used. Participants also listed characteristics of other group members they thought were important for the group to be successful and were asked to explain why such characteristics are important. Finally, the survey included an open-ended question asking for any additional thoughts about study groups.

## 6. Results

### 6.1. Beliefs, Frequency of Collaboration, and Preferences

**Figure 1:**
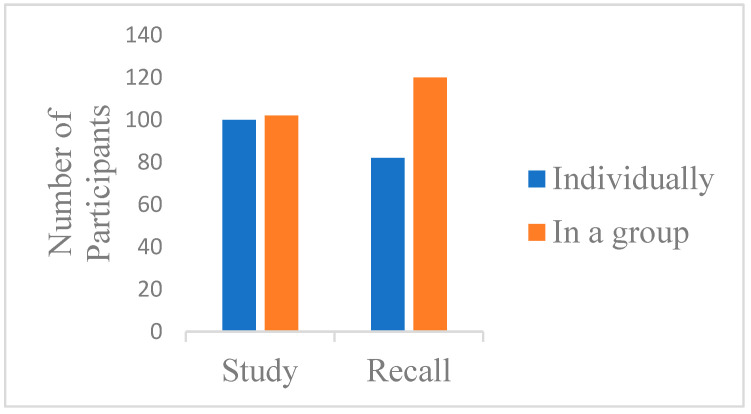
Which do you think is more effective—studying in groups or studying individually? Which do you think is more effective—recalling in groups or recalling individually?

We used chi-square tests to investigate students’ beliefs and preferences about studying. Firstly, when asked if they believed whether studying in a group or studying individually was more effective, an equal number of participants chose each option. As shown in Figure 1, there was no significant difference in the number of students who believed that studying in groups or individually was more effective, *χ*^2^(1, n = 202) = 0.02, *p* = 0.89. However, when asked about recalling in groups, more students believed that it was more effective to recall in a group than to recall information individually, *χ*^2^(1, n = 202) = 6.42, *p* = 0.01.

**Figure 2:**
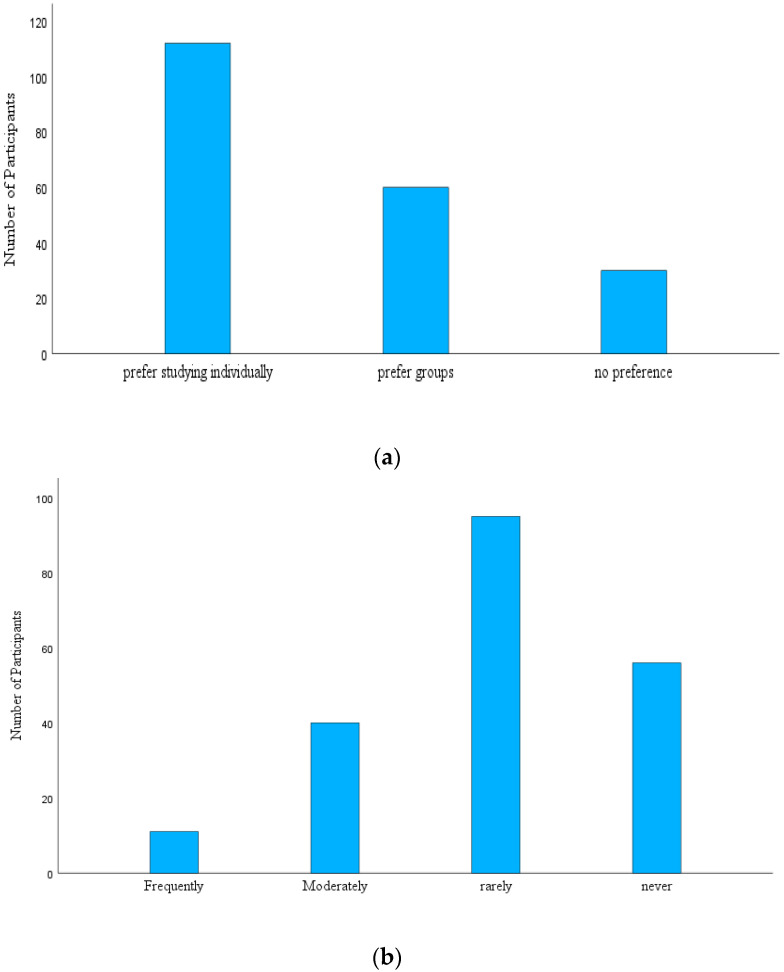
(**a**) Do you prefer to study for exams in a group or individually? (**b**) How often do you use study groups?

Surprisingly, we found that although most students believed group study to be equally effective and group recall to be more effective than individual recall, most students preferred to study individually for their exams (see Figure 2a), *χ*^2^(2, n = 202) = 51.13, *p* < 0.001.

Lastly, when students were asked whether they used study groups, many of them reported that they rarely use study groups (see Figure 2b), *χ*^2^(3, n = 202) = 72.89, *p* < 0.001. These findings suggest that students believe recalling in study groups is beneficial, but they prefer to study individually and rarely use study groups, indicating their actual and preferred study behaviors are not consistent with their beliefs.

### 6.2. Group Recall and Group Studying

We then analyzed the responses to the questions “Why do you think studying in groups/individually is more effective?” and “Why do you think recalling in groups/individually is more effective?”. Participants’ responses were coded into different categories adapted from Rybczynski and Schussler [26] or constructed locally by following the best practices for qualitative data coding outlined by Syed and Nelson [34]. For example, many students wrote “because I want to learn from other students”; we categorized these responses as social learning. The first 30 percent of responses were coded by two persons, and interrater reliability was calculated using Cohen’s kappa in SPSS to ensure that the reliability of each column was above 60% [35]. After interrater reliability was high, one of the coders finished the rest of the responses.

**Figure 3:**
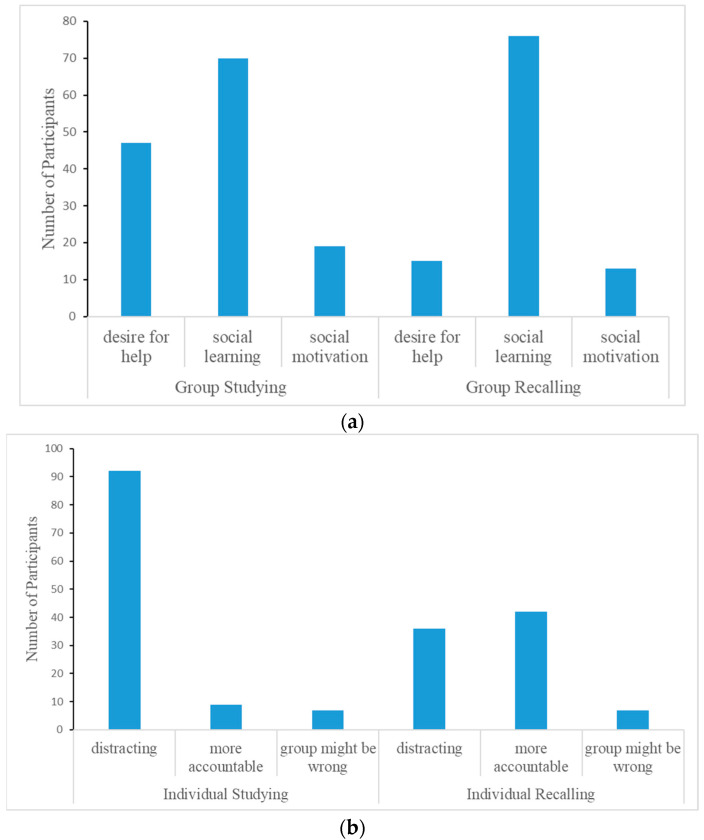
(**a**) Distribution of responses to why preferring studying in groups/individually. (**b**) Distribution of responses to why preferring recalling in groups/individually.

As shown in Figure 3a,b, there were three main reasons why participants preferred to study/recall in groups: (1) desire for help—to obtain help from other members, (2) social learning—to learn from other members, and (3) social motivation—work hard due to social pressure. Figure 3a,b also shows the three main reasons why participants prefer to study/recall individually: (1) less distraction—groups are distracting, (2) more accountable—to make sure individuals master the materials instead of hearing answers from other members, and (3) misinformation—individuals were worried about learning incorrect information from other members. These responses underscore that participants interpreted our questions of “effective” strategies as strategies that are effective for learning.

We compared the distribution of their responses in Figure 3a and Figure 3b using chi-square tests for independence. We report *Cramér’s V* as a measure of effect size. When interpreting *Cramér’s V* for the chi-square test for independence when df* = 1, a small effect size is 0.1, a medium effect size is 0.3, and a large effect size is 0.5 or greater [36,37]. We found that the distribution of studying and recalling in groups was different, *χ*^2^(2, 240) = 13.87, *p* < 0.001, *Cramér’s V* = 0.24, and the distribution of studying and recalling individually was different, *χ*^2^(2, 193) = 43.73, *p* < 0.001, *Cramér’s V* = 0.48. Specifically, the main reason for choosing to both study/recall in groups is social learning. However, compared to recalling in groups, more participants choose to study in groups because they desire help. Regarding the primary reasons for studying/recalling individually, most students want to study individually because study groups are distracting. Many students also want to recall individually because groups are distracting, but this is not the dominant reason for recalling individually. Rather, many students want to recall individually because they are more accountable; they want to ensure they can retrieve information individually.

### 6.3. Strategies Used When Studying Individually and in Groups

Table 1a,b shows the strategies students listed when studying individually and in groups. Looking first at the strategies reported when students study individually, we found that 69.8% of participants reported they re-read notes and textbooks, and the mean rank was high, indicating a large number of students use re-reading materials frequently. Also, many students (over 20%) reported they use flashcards, do practice problems, do practice recall/Quizlet, use review sheets, re-write notes, and memorize/repeat materials.

Turning next to the strategies reported when students study in groups, over 40% of students reported that they teach each other/discuss and quiz each other in groups. Over 20% of students reported that they ask for help/answers and compare notes with group members. Notably, these strategies were never listed as strategies used by individuals. However, the rest of the strategies listed for group study were also listed for individual study, but they were listed less frequently. Therefore, these results demonstrate that the strategies students use when studying in groups/individually are generally consistent but that participants prioritize interactive strategies.

### 6.4. Characteristics of Other Group Members

Table 2 displays the important characteristics of other group members listed by participants from the most reported to the least reported (more than five cases). More than 50% of participants claimed that “focused/task-oriented” is an important characteristic of other group members. This corresponds to the previous findings that lacking focus is one of the biggest concerns of groups [26]. In addition, more than 30% of participants reported the characteristics responsible/hard working (39.1%) and motivated/desire to participate (35.1%). More than 20% of participants thought being cooperative/flexible was important (23.8%). More than 10% of participants hoped their group members could have some positive attributes, such as being helpful/patient (19.8%), kind/considerate (13.9%), and easy-going/fun/talkative (10.9%). Building on these findings, many students think characteristics that can help complete tasks are important, and some students also think having positive traits is important for their group members.

### 6.5. Differences Between Students Who Frequently and Rarely Use Study Groups

We split our participants into two groups—“frequently” or “moderately” used groups and “rarely” or “never” used groups. By comparing these groups’ studying and recalling choices, preferences, preferred strategies when studying individually and in groups, and favored characteristics of other group members, we can investigate how previous experience with groups is related to students’ metacognitive beliefs. Again, we compared the distribution of their responses using chi-square tests for independence. Because this required six chi-square tests, we adopted 0.008 as the significant p value instead of 0.05 in order to correct for type 1 error.

Table 3 shows beliefs and preferences for students who frequently/moderately use study groups and students who rarely/never use study groups. More students who frequently/moderately use study groups think studying in groups is more beneficial, but more students who rarely/never use study groups think studying individually is more beneficial, *χ*^2^(1, 202) = 15.74, *p* < 0.001, *Cramér’s V* = 0.28. This result indicates that students’ previous group study experience is related to their studying beliefs and qualifies our previous finding that there was no significant difference in studying beliefs when we combined students who often use and rarely use study groups. Importantly, however, previous group experience had no impact on the perceived benefits of collaborative recall; more students believe recalling in groups to be more effective regardless of whether they previously use study groups or not, *χ*^2^(1, 202) = 3.54, *p* = 0.06, *Cramér’s V* = 0.13. We also found that previous study group experiences are related to study preferences. Specifically, as shown in Table 3, more students in the frequently/moderately used group prefer to study for exams in groups, while more students in the rarely/never group prefer to study for exams individually, *χ*^2^(1, 172) = 31.39, *p* < 0.001, *Cramér’s V* = 0.43.

In addition, we examined whether group use experiences are related to strategies students use when studying in groups and individually. We only included the top six strategies for analysis because we needed to ensure that there were enough cases in each cell for analysis. Previous group use experiences do not correlate with strategies students use in groups, *χ*^2^(5, 340) = 3.44, *p* = 0.63, *Cramér’s V* = 0.10 and individually, *χ*^2^(5, 460) = 2.24, *p* = 0.82, *Cramér’s V* = 0.07, suggesting most frequently used strategies are not influenced by students’ group experiences.

Last, we examined whether group use experiences are related to students’ favored characteristics of other group members. Again, we picked the 5 top characteristics because we needed to make sure there were enough free report responses in each cell for the chi-square analysis. More students in the rarely/never group reported characteristics that can help achieve their goals, such as focused, motivated, and reliable *χ*^2^(4, 352) = 22.68, *p* < 0.001, *Cramér’s V* = 0.25. This indicated that more students who rarely or never use study groups like group members who can achieve goals, but students who frequently or moderately use study groups also hope other members are helpful and flexible.

## 7. Discussion

The present study investigated students’ beliefs about study groups and further characterized the reasons for their beliefs. We were also interested in how students’ beliefs corresponded to why they want to study/recall in groups, strategies they use individually and in groups, and important characteristics of other group members.

### 7.1. Beliefs, Frequency of Collaboration, and Preferences

In the current study, more students believed *recalling* in groups is more effective than recalling individually; however, there was no significant difference in the number of students who believed *studying* in groups was more effective than studying individually. These findings indicate that more students have a positive attitude toward group recalling, which is consistent with previous findings [25,26,27,29]. However, when studying for exams, more students prefer to study individually [29]. Additionally, nearly half of our participants rarely use study groups, and many of them never use study groups. Thus, metacognition about group studying is not consistent with real or preferred studying behaviors [19]. In general, students think study groups are beneficial, but they prefer to study individually.

Previous experience in collaborative groups influenced these conclusions to some extent. Those frequently using study groups believe group study to be more effective and prefer to study for exams in groups. In contrast, previous experience had no impact on students’ beliefs that collaborative recall was effective. More generally, students’ beliefs and preferences are related to their frequencies of using study groups.

### 7.2. Group Recall and Group Studying

Wissman and Rawson [25] reported that individual studying is thought to be more effective, but group testing is regarded as more helpful. We partially replicated their findings by showing that there was no significant difference in the number of students who believe studying individually or in groups is more effective, but more students believe recalling in groups is more effective. In the current study, we found that nearly 50% of students believe studying in groups is more effective, whereas only 21% of Wissman and Rawson’s participants thought so. We used an online chi-square calculator [38] to compare the frequency data in the current study with those in Wissman and Rawson and found that the percentage in the current study is significantly higher (*p* < 0.00). Given that Wissman and Rawson did not ask participants why they thought studying individually was more effective, the difference could be ascribed to students’ increasing awareness of the advantages of group study. Additional explanations for the discrepancy between studies include the use of different samples and possible time of measurement effects; COVID-19 could impact student perceptions of learning and collaboration.

We also extended Wissman and Rawson’s [25] findings by asking students why they believe studying/recalling individually/in groups is more effective using open-ended questions. Specifically, compared to studying in groups, fewer students desire others’ help when recalling in groups. The predominant reason for studying individually is that groups are distracting, while the main reason for recalling individually is to make sure individuals can recall the information in the future instead of group members telling them the answer. These data are relevant to theories of self-regulated learning because they characterize students’ beliefs about why they seek out collaborative study/recall or individual study/recall to regulate their learning. Further, because students believe studying and recalling are different processes, experimenters and instructors should give students more specific instructions in the future instead of using a general term, such as “learning”.

### 7.3. Strategies Used When Studying Individually and in Groups

Replicating previous research [20], most students like to re-read their notes and textbooks when studying individually. In addition, many students also reported that they do practice problems, use flashcards, make review sheets, and practice recall, all of which is consistent with Karpicke et al.’s study. The only difference in the current study was that many students reported that they would like to watch videos and use some other online tools, such as YouTube. This could be attributed to the development and accessibility of online technologies, as well as the reliance on online courses due to COVID-19. The most novel findings from our strategy questions involve collaborative groups. Importantly, when studying in groups, more students prefer to teach each other/discuss, quiz each other, and ask for help. That is, when studying in groups, students are more likely to use strategies emphasizing collaboration and interaction. These findings are conceptually consistent with previous studies [25,26,29] in demonstrating that collaborative groups frequently quiz each other and compare and share notes.

Interestingly, we found that previous experience in collaborative groups had no impact on preferred strategies. This finding does not align with McCabe and Lummis [29] claiming that students with more study group experiences use more effective strategies. The disagreement between findings can be ascribed to the design of our study. In their study, the authors listed all the strategies, and students could rate the frequency of each one, but we asked our participants to list strategies freely. It is possible that students judge the frequency of using various strategies differently when they are given choices or are asked to list freely.

### 7.4. Characteristics of Other Group Members

More than half of students hope their group members are focused, which supports the findings of Rybczynski and Schussler [26] that individuals think lack of focus is one of the biggest problems of study groups. This finding is also conceptually consistent with collaborative memory research that demonstrates group composition can influence group performance [39,40]. Many participants reported that they did not want their groups to go off-topic. Additionally, many students prefer task-oriented characteristics, including being motivated and responsible. Some students also prefer their group members to be talkative, cooperative, and helpful. These characteristics can provide individuals with a supportive and encouraging environment in which they collaborate. Therefore, many participants think their group members should have some characteristics that are important to achieve their goals, and some hope their group members are supportive and positive. It is worth noting that students frequently/moderately using study groups also emphasize the helpfulness and flexibility of their group members, while those rarely/never using study groups only highlight goal-relevant characteristics.

### 7.5. Theoretical and Practical Implications

This study has important practical and theoretical implications for self-regulated learning. Self-regulated learning requires students to plan for their learning, monitor their on-going learning, and adjust their study behavior if necessary. There are many theories and models regarding the relationship between these elements of self-regulated learning [16,41]. According to the results of the present study, students may consider the unique strategies afforded by collaboration when regulating their own learning. For example, when studying in groups, the current participants prioritized interactive strategies that allow them to clarify information and quiz each other to test how much information each group member has mastered. Our participants also reported nuanced beliefs about how collaboration can help or hurt their learning during study and retrieval and how group composition affects learning. Given these findings, there is a great need for theories of metacognition to include collaboration as a potential factor that may influence how students’ monitor and regulate their own learning. Toward this end, the present study can serve as a starting point for investigating how students’ beliefs about collaborative study, collaborative recall, and group composition affect their use of study groups, their learning preferences, and how they monitor and adapt their learning processes.

It is important that educators understand students’ concerns about study groups so they can help students better use study groups in educational contexts. Also, students can learn that they may be underusing study groups. Specifically, although students believe study groups are beneficial—and in many cases, study groups *are* beneficial—they typically adhere to the traditional studying method. Students may, therefore, consider using study groups more frequently and examine their effectiveness. Taken altogether, our findings can be used to equip educators and students with knowledge about how to better incorporate study groups into their own educational practices.

## 8. Limitations and Future Directions

We acknowledge several limitations in the current study. The sample was collected from one university at a single point in time. As such, it is important to replicate the results across a wider range of participants to demonstrate generalizability, especially when COVID-19 influenced the way students are educated to a large extent [30,31,32]. Future research can examine students’ metacognition about collaboration again after the pandemic to determine any lasting effects of the pandemic on metacognitive beliefs about collaboration. Further, the questionnaire was aimed at collaborative learning generally rather than focusing on the role of collaborative learning for specific class topics. Further research is necessary to determine if the results presented here generalize across different content and materials. Also, we asked students which strategies are “effective” [33], which could be interpreted in different ways (e.g., improving their grades, assisting in their long-term learning, or enhancing their short-term performance). Answers to our “why” questions suggested that students interpret it as effective for learning. However, future research can examine more precisely which aspects of learning students believe are most influenced by collaboration.

Further research is necessary to follow up on and extend some of the key findings from the present study. The results of this study suggest that there is a discrepancy between students’ beliefs about study groups and students’ learning preferences. That is, students seem to realize that study group is beneficial but prefer to study individually for exams. Building on this inconsistency between metacognition and learning behavior, further research can probe why this discrepancy occurs and whether an intervention of some kind can help students voluntarily adopt the use of study groups to improve their performance. Students’ beliefs about studying in groups or individually are related to their group study experiences. Future research can investigate why previous study group experiences are related to studying beliefs and preferences. Specifically, researchers can examine if previous experiences are the cause or the results of their beliefs and preferences. Or there may be a third factor that can influence students’ group use experiences and beliefs. For example, extraverted students may believe study groups are more beneficial and may even benefit more from studying groups than their introverted counterparts.

## 9. Conclusions

In conclusion, this study investigated students’ metacognition about group study and showed that students’ learning activity is inconsistent with their attitudes towards group studying. Namely, students infrequently use study groups, choosing instead to study individually, even though they recognize the general benefits of group study. Their beliefs and preferences are related to their frequencies of using study groups. We think such a discrepancy is important, from both a theoretical and practical standpoint, and is a worthy topic for future research, such as factors that discourage students from study groups, the application of collaborative recall in classrooms, and instructors’ impact on adjusting students’ metacognition and preferences.

## Figures and Tables

**Table 1:** (**a**) Strategies students use when studying individually. (**b**) Strategies students used when studying in groups.

(**a**)					
Strategy	Percent who list strategy		Percent who rank as #1 strategy		Mean rank
Re-reading notes and textbooks	69.8	(141)	32.7	(66)	1.8
Flashcards	35.6	(72)	10.4	(21)	2.3
Do practice questions	34.1	(69)	11.9	(21)	2.1
Practice recall/Quizlet	31.2	(63)	12.9	(26)	2.0
Watching videos/outside resources	28.7	(58)	5.9	(12)	2.5
Review sheet	27.7	(56)	8.9	(18)	2.3
Re-write notes	20.8	(42)	9.9	(20)	1.8
Memorize/repetition	20.3	(41)	8.9	(18)	2.0
Focus on wrong answers/unclear information	9.9	(20)	0.5	(1)	2.9
Mnemonics/association	6.9	(14)	1.5	(3)	2.4
Highlight (in notes/books)	5.9	(12)	1.5	(3)	2.2
Create a good environment	5.9	(12)	3.5	(7)	1.6
Spaced repetition/interleaving	4.0	(8)	1.0	(2)	2
Think of real life examples	2.5	(5)	0.5	(1)	2.8
(**b**)					
Strategy	Percent who list strategy		Percent who rank as #1 strategy		Mean rank
Teach each other/discussion	44.1	(89)	21.2	(43)	1.7
Quiz each other	40.1	(82)	13.9	(28)	2.0
Ask for help/answers	22.3	(45)	10.4	(21)	1.8
Flashcards	20.8	(42)	9.4	(19)	1.9
Compare answers/notes	20.3	(41)	7.4	(15)	2.0
Re-reading notes/textbooks	20.3	(41)	5.9	(12)	2.3
Do practice problems	18.3	(37)	9.4	(19)	1.8
Make review sheets/study guide	18.3	(37)	9.9	(20)	1.6
Practice recall/Quizlet	8.9	(18)	2.5	(5)	2.3
Focus on wrong answers	7.9	(16)	3.0	(6)	1.9
Re-write notes	7.9	(16)	1.0	(2)	2.8
Watch videos/ask professors/outside resources	6.4	(13)	1.5	(3)	2.8
Group focuses on one topic	4.0	(10)	1.0	(4)	2
Mnemonics/association	3.5	(7)	1.0	(2)	2.3

Note. The numbers in parentheses reflect the raw participant numbers.

**Table 2:** Important characteristics of other group members.

Characteristics	Percent Who List Strategy	
Focused/task oriented	56.4	(114)
Responsible/hard-working/reliable	39.1	(79)
Motivated/desire to participate	35.1	(71)
Flexible/receptive/cooperative	23.8	(48)
Helpful/patient	19.8	(40)
Kind/considerate	13.9	(28)
Easy-going/talkative/fun	10.9	(22)
Communication skills/organized	8.9	(18)
Have similar goals	5.4	(11)
Humble/willing to make mistakes	5.4	(11)
Similar knowledge or skills	5.0	(10)
Similar/equal contribution	4.5	(9)
Leadership	4.0	(8)
Skeptical/critical	3.0	(6)

Note. The numbers in parentheses reflect the raw participant number.

**Table 3:** Beliefs and preferences for students who frequently/moderately use study groups vs. students who rarely/never use study groups.

		Frequently/Moderately Group	Rarely/Never group
Beliefs about studying	Study in groups is more effective	38	64
	Study individually is more effective	13	87
Beliefs about recalling	Recalling in groups is more effective	36	84
	Recalling individually is more effective	15	67
Studying preference	Study in groups for exams	32	28
	Study individually for exams	15	97
Favored characteristics of other group members	Focused	26	114
	Motivated	17	71
	Reliable	22	79
	Flexible	23	48
	Helpful	22	40

Note: some students reported no studying preference.

## Data Availability

Data for this experiment are publicly available on OSF: https://osf.io/3fx4d/?view_only=6510a49c0d874f7999d70c19c56a869c (accessed on 1 July 2022).

## Appendix A

1. How often do you use study groups?
  Frequently (once or more per week)
  Moderately (once or twice per month)
  Rarely (once or twice per semester)
  Never
2. Which do you think is more effective—studying in groups or studying individually?
  Why do you think it is more effective?
3. Which do you think is more effective-- recalling (remembering) information in groups or recalling information individually?
  Why do you think it is more effective?
4. Do you prefer to study for exams in a group or individually?
  Why do you prefer to study this way?
5. List the strategies you use when studying individually and rank the strategies in terms of how frequently you use them (the first strategy you list should be the one you use most frequently; the last strategy you list should be the one you use least frequently). (e.g., 1, XXX 2, XXX 3, XXX 4, XXX 5, XXX)
6. List the strategies you use when studying in a group and rank the strategies in terms of how frequently you use them (the first strategy you list should be the one you use most frequently; the last strategy you list should be the one you use least frequently). (e.g., 1, XXX 2, XXX 3, XXX 4, XXX 5, XXX)
7. What characteristics of other group members do you think are important in order for study groups to be productive? (e.g., XXX, XXX, XXX, XXX…)
  Why do you think these characteristics are important?
8. Do you have any other thoughts regarding study groups that you would like to share?
9. Your age (in years): __________
10. Gender:
  Male
  Female
  Prefer not to say
  Other: ____________
11. Current year in school:
  First year college
  Second year college
  Third year college
  Fourth year college
  Other: _____________
12. Ethnicity:
  Hispanic or Latino
  Not Hispanic or Latino
13. Race:
  American Indian/Alaska Native
  Asian
  Black/African American
  Native Hawaiian/Other Pacific Islander
  White
  Prefer not to say
  Other:___________
